# Torquetenovirus Loads in Peripheral Blood Predict Both the Humoral and Cell-Mediated Responses to SARS-CoV-2 Elicited by the mRNA Vaccine in Liver Transplant Recipients

**DOI:** 10.3390/vaccines11111656

**Published:** 2023-10-28

**Authors:** Claudia Minosse, Giulia Matusali, Silvia Meschi, Germana Grassi, Massimo Francalancia, Gianpiero D’Offizi, Pietro Giorgio Spezia, Anna Rosa Garbuglia, Marzia Montalbano, Daniele Focosi, Enrico Girardi, Francesco Vaia, Giuseppe Maria Ettorre, Fabrizio Maggi

**Affiliations:** 1 Laboratory of Virology, National Institute for Infectious Diseases Lazzaro Spallanzani IRCCS, Via Portuense 292, 00149 Rome, Italy; claudia.minosse@inmi.it (C.M.); giulia.matusali@inmi.it (G.M.); massimo.francalancia@inmi.it (M.F.); pietro.spezia@inmi.it (P.G.S.); annarosa.garbuglia@inmi.it (A.R.G.); fabrizio.maggi@inmi.it (F.M.); 2 Laboratory of Cellular Immunology and Pharmacology, National Institute for Infectious Diseases Lazzaro Spallanzani IRCCS, Via Portuense 292, 00149 Rome, Italy; germana.grassi@inmi.it; 3 Department of Liver Transplantation POIT, Clinical and Research Department of Infectious Diseases, National Institute for Infectious Diseases Lazzaro Spallanzani IRCCS, Via Portuense 292, 00149 Rome, Italy; gianpiero.doffizi@inmi.it (G.D.); marzia.montalbano@inmi.it (M.M.); giuseppe.ettorre@inmi.it (G.M.E.); 4 North-Western Tuscany Blood Bank, Pisa University Hospital, 56124 Pisa, Italy; daniele.focosi@gmail.com; 5 Scientific Direction, National Institute for Infectious Diseases Lazzaro Spallanzani IRCCS, Via Portuense 292, 00149 Rome, Italy; enrico.girardi@inmi.it; 6 General Direction, National Institute for Infectious Diseases Lazzaro Spallanzani IRCCS, Via Portuense 292, 00149 Rome, Italy; francesco.vaia@inmi.it

**Keywords:** TTV, COVID-19, vaccination, anti-spike antibodies, neutralizing antibodies, liver transplantation

## Abstract

Three years into the COVID-19 pandemic, mass vaccination campaigns have largely controlled the disease burden but have not prevented virus circulation. Unfortunately, many immunocompromised patients have failed to mount protective immune responses after repeated vaccinations, and liver transplant recipients are no exception. Across different solid organ transplant populations, the plasma levels of Torquetenovirus (TTV), an orphan and ubiquitous human virus under control of the immune system, have been shown to predict the antibody response after COVID-19 vaccinations. We show here a single-institution experience with TTV viremia in 134 liver transplant recipients at their first or third dose. We found that TTV viremia before the first and third vaccine doses predicts serum anti-SARS-CoV-2 Spike receptor-binding domain (RBD) IgG levels measured 2–4 weeks after the second or third dose. Pre-vaccine TTV loads were also associated with peripheral blood anti-SARS-CoV-2 cell-mediated immunity but not with serum SARS-CoV-2 neutralizing antibody titers.

## 1. Introduction

Liver transplantation requires chronic maintenance of immunosuppression in most patients to prevent graft rejection [1]. As such, liver transplant recipients are left immunocompromised and have a clearly increased risk of infections (viral or other pathogens), which is greatest in the first six months after transplantation when patients are profoundly immunosuppressed [2]. Thus, vaccine-preventable infections may have severe consequences in liver transplant recipients, who show higher morbidity and mortality with respect to the general population. In addition, due to strong immunosuppression, poor vaccine response is often found and extensive vaccination schedules are recommended for liver transplant recipients [3,4,5,6,7,8,9,10]. COVID-19 represents a significant cause of mortality and morbidity in liver transplant recipients [11] and immunocompromised patients, and a different model of response to COVID-19 vaccination has been reported across different solid organ transplant cohorts [12,13,14,15].

In many different types of solid organ transplants, the ubiquitous non-pathogenic Torquetenovirus (TTV), the prototype of a large group of circular, single-stranded DNA viruses belonging to the Anelloviridae family, has been exploited as a marker of functional immune competence [16,17]. TTV has several intriguing characteristics, including a small genome that makes the replication-competent virus genetically simpler, a high level of genetic variability with a consistent number of species and genotypes hitherto known, and an astonishing ability to induce persistent infections in the host with a wide range of viremia levels [18]. Furthermore, TTV is the most abundant component in the normal mammalian virome [19]. More specifically, the measure of the TTV copy number in peripheral blood has been associated with the degree of immunosuppression of the host [20,21], and viremia-driven optimization of immunosuppressive drugs is being attempted to minimize the risk of infection and rejection, thereby prolonging patient and graft survival [22,23,24,25,26,27]. Recently, TTV viremia measurement before vaccination has been shown to predict COVID-19 vaccine antibody responses in lung transplant recipients and kidney transplant recipients [28], thus suggesting the utility of the anellovirus TTV as a biomarker for vaccine immunogenicity and efficacy. Here, we investigated whether the predictive value of TTV viremia is also valid in liver transplant recipients subjected to consecutive doses of mRNA SARS-CoV-2 vaccines.

## 2. Materials and Methods

### 2.1. Study Design and Population

This study was conducted on liver transplant recipients who were followed up at the “Lazzaro Spallanzani” National Institute for Infectious Diseases (INMI) in Rome within the VAX-TRA study. Inclusion criteria were having 2 or 3 doses of a COVID-19 mRNA vaccine (BNT162b2 or mRNA-1273) and having available serum samples collected on the day of the first (T0) or third vaccine dose (T3). Patients with a documented former SARS-CoV-2 infection were excluded to minimize confounders. In total, 126 of the liver transplant recipients had antibody responses assessed after the 2nd vaccine dose and 43 after the 3rd dose. All participants provided informed consent. The study was approved by the Comitato Etico Territoriale Area 4 (approval number: DR n°G01659-10 February 2023; amendment adopted with no. 4-2023 CET Registry).

### 2.2. TTV DNA Quantification

Viral DNA extraction from serum samples was carried out using the Qiasymphony platform (Qiagen, Hilden, Germany). The TTV viral load (VL) was measured in the serum collected at T0 and T3, using the CE-IVD-marked TTV R-GENE^®^ kit (bioMérieux, Marcy-l’Etoile, France). This assay uses 5′-nuclease TaqMan technology, is commercialized in the format of a ready-to-use amplification mixture, and allows the amplification of a 128 bp fragment of a 5′ untranslated region (UTR) of the TTV genome. Amplification was performed using the Rotor-Gene Q 2plex (Qiagen, Hilden, Germany) according to the manufacturers’ instructions. The TTV R-GENE^®^ kit provides standards to generate a quantification curve with a dynamic range of 10^1^ to 10^9^ copies/mL, allowing the measurement of TTV VL values in copies/mL. The results are expressed in log copies/mL. In each amplification, a negative control was included. The technique shows high sensitivity and is capable of detecting TTV DNA copies in blood at levels as low as 1.1 log. Undetected TTV VL were arbitrarily assigned a value of 1.0 log. We have previously described the procedures here employed for the determination of copy numbers, specificity, sensitivity, intra-assay and inter-assay precision, and reproducibility [29,30]. The measured overall inter-assay variation was <0.5 log.

### 2.3. SARS-CoV-2 IgG Antibody Testing

Anti-SARS-CoV-2 Spike receptor-binding domain (RBD) IgG testing was performed in serum samples collected 2–4 weeks after the second or third dose of vaccines using the Abbott Architect SARS-CoV-2 IgG II Quant assay (Abbott, Chicago, IL, USA). To convert antibody levels into binding antibody units (BAU)/mL, a multiplication factor of 0.142, adapted to the World Health Organization standard for SARS-CoV-2 immunoglobulin, was applied. As described in the manufacturer’s instructions, the anti-RBD IgG titers are measured on a scale of 7.1 (as a positive cut-off) to 11,360 BAU/mL (as an upper threshold of quantification). Thus, all patients who displayed anti-RBD IgG titers ≥ 7.1 BAU/mL were classified as vaccine responders; seronegative patients (IgG titers < 7.1 BAU/mL) were categorized as non-responders.

### 2.4. SARS-CoV-2 Microneutralization Assay

The microneutralization assay (MNA) was performed using the Wuhan-D614G strain (GISAID accession ID EPI_ISL_568579). Starting from a 1:10 dilution of serum samples inactivated at 56 °C for 30 min, twofold serial dilutions were titrated in duplicate. Equal volume of serum dilutions and 100 TCID50 of SARS-CoV-2 were mixed and incubated at 37 °C, 5% CO_2_ for 30 min. Virus/serum mixtures (100 μL) were then added to Vero E6 cell monolayers in 96-well tissue culture plates. After 48 h at 37 °C and 5% CO_2_, cytopathic effect (CPE) was estimated by observation at light microscope. Neutralization titer (MNA90) was defined as the serum dilution inhibiting ≥90% of the CPE. Serial dilutions ranging from 1:10 to 1:1280 were performed. Neutralization titers equal to or greater than 1:10 were considered positive.

### 2.5. Interferon-Gamma ELISA Assay

Interferon-gamma (IFN-γ) production was evaluated in response to Spike protein stimulation [31]. Peripheral blood was collected in heparin tubes and stimulated, or not, with a pool of peptides spanning the Spike protein (Prot-S code 130126701; Prot-S1 code 130127048; Prot-S+ code 130127312; Miltenyi Biotech, Bergisch Gladbach, Germany) at 37 °C (5% CO_2_). The spontaneous cytokines’ release was calculated in an unstimulated culture, and a superantigen (SEB) was used as a positive control. After 16–20 h of stimulation, plasma samples were harvested and stored at −80 °C. IFN-γ production was quantified in the plasma samples using an automatic ELISA (ELLA™, Bio-Techne, Minneapolis, MN, USA). The detection limit of the assay was 0.17 pg/mL and positive IFN-γ response was defined as ≥12 pg/mL.

### 2.6. Statistical Analysis

Variables were expressed as follows: (i) continuous variable as mean (standard deviation), median, and interquartile range (25th percentile; 75th percentile); (ii) categorical data as frequencies and percentages. The non-parametric Mann–Whitney U-test was used to compare the continuous variables. The distribution of categorical variables was compared using the Fisher’s exact test and the Chi-square test. Correlation analyses between two continuous variables were assessed using non-parametric Spearman tests. Paired data were compared using the Wilcoxon test. All the tests were two-tailed and considered statistically significant when the *p*-value was <0.05. All the above analyses were performed using Prism 8.0.2. Receiver operating characteristic (ROC) curve analyses were carried out for antibody response after two or three doses using the MedCalc software. Multivariable logistic regression was performed using SPSS 28.0 (IBM Statistics 28.0 version) to identify independent predictors of antibody response.

## 3. Results

### 3.1. Study Population

A total of 169 serum samples were obtained. Of these, 126 were from liver transplant recipients with anti-SARS-CoV-2 antibody response assessed after the second vaccine dose (recruited from 22 March to 22 April 2021), and 43 were from liver transplant recipients with antibody response assessed after the third vaccine dose (recruited from 20 September to 24 September 2021). Demographic and laboratory characteristics of the study population are shown in Table 1. No participant had a history of prior SARS-CoV-2 infection, as confirmed serologically by negative anti-SARS-CoV-2 Nucleoprotein IgG before T0 and T3 and negative anti-SARS-CoV-2 Spike-RBD IgG before T0. The mean age of the patients was 63.2 years (standard deviation: 9.3 years) and 63.6 years (standard deviation: 7.7 years) in individuals who received two or three vaccine doses, respectively. Gender distribution is as follows: 81.8% males and 18.2% females in the group who received two doses of vaccine, and 88.4% males and 11.6% females in the group who received three doses of vaccine.

After two doses of vaccine, 99/126 (78.6%) liver transplant recipients displayed positive anti-RBD IgG; after the third dose, 41/43 (95.3%) liver transplant recipients presented positive anti-RBD IgG. The median antibody level was 2.1 log BAU/mL after two doses of vaccine, which significantly increased by 1.3 log after the third dose.

### 3.2. TTV VL and Anti-SARS-CoV-2 RBD IgG Response

TTV VL was measured in serum samples collected at T0 and T3. As shown in Table 1, 167 out of 169 serum samples (98.8%) were TTV positive, with a total VL of 4.8 log copies/mL in the median. No significant differences in TTV prevalence or median VL were observed between T0 and T3. Table 2 shows patient characteristics and clinical parameters grouped by anti-SARS-CoV-2 seroconversion after two or three vaccine doses. 

**Figure 1:**
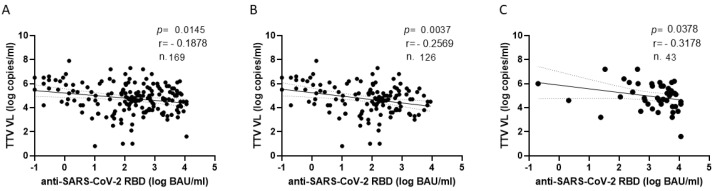
Correlation between baseline TTV VL and post-vaccination levels of anti-SARS-CoV-2 antibodies when analyzed totally (**A**) and after second (**B**) or third vaccine dose (**C**).

Before the first two vaccine doses (i.e., at T0), TTV VL was 1 log higher in non-responders’ than in responders’ sera (5.6 vs. 4.6 log copies/mL, *p* < 0.0001). This significant association between pre-vaccine TTV VL and the size of the anti-RBD IgG response was not observed with the third dose of vaccine. The correlation between TTV VL and serum anti-RBD IgG levels was then evaluated. As shown in Figure 1, a significant inverse correlation was observed between pre-vaccination TTV VL and levels of serum anti-SARS-CoV-2 RBD IgG. This correlation was evident when all samples were considered (r = −0.187; *p* = 0.014; Figure 1A), or in subgroups by the number of vaccine doses (r = −0.256; *p* = 0.003 and r = −0.317; *p* = 0.037 after two or three doses, respectively; Figure 1B,C).

No significant variation in TTV load was observed in individual patients: in 35 patients, with serum available at T0 and T3, the median of TTV load was 5.0 (IQR 4.2–5.6) and 4.7 (IQR 4.1–5.8), respectively, at T0 and T3, with a *p* = 0.9612 (calculated with Wilcoxon test). Considering all 169 serum samples, the median of TTV load is not significantly different between females and males (4.6; IQR 4.2–5.2 and 4.9; IQR 4.0–5.6, respectively; *p* = 0.4605), and between patients with BMI < 30 or BMI > 30 (4.8; IQR 4.0–5.6 and 4.7; IQR 4.1–5.9, respectively; *p* = 0.8893). There is no correlation between TTV load and age (r = −0.0324; *p* = 0.6756) when including all serum samples.

**Figure 2:**
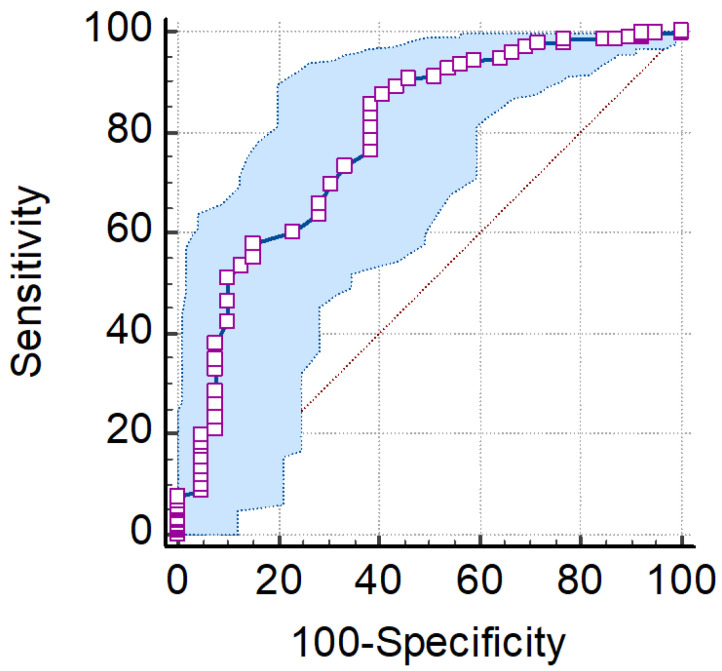
Receiver operating characteristic (ROC) curve for distinguishing the optimal cut-off value for TTV VL.

**Figure 3:**
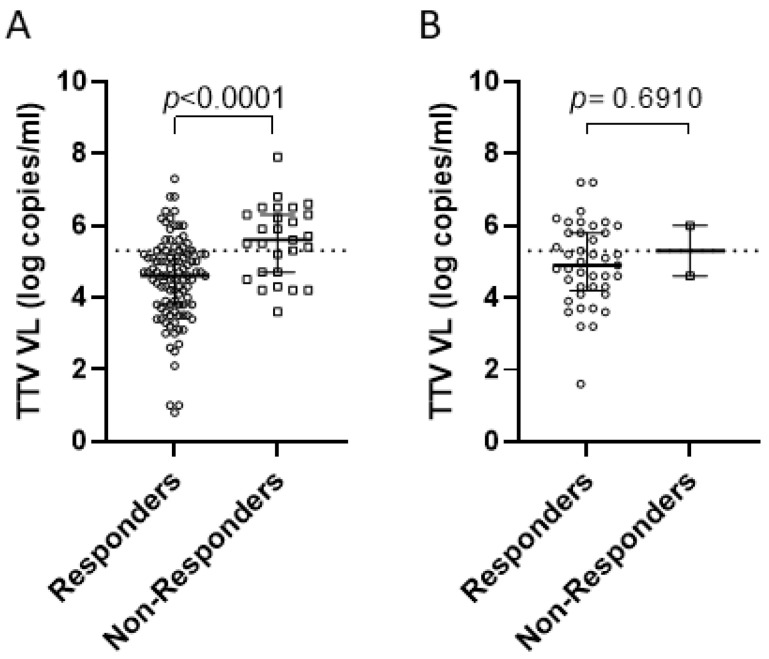
TTV VL and anti-SARS-CoV-2 IgG response to second (**A**) and third (**B**) vaccine dose. TTV VL of responder (○) and non-responder (□) patients were reported. Median, upper, and lower quartiles are represented by horizontal lines. Dotted line indicates the predictive threshold of 5.3 log copies/mL.

It is difficult to establish with certainty the serum TTV VL threshold above which the probability of experiencing a vaccine response is the highest. However, the optimal cut-off value for serum TTV load was determined at 5.3 log DNA copies/mL (sensitivity: 75.7%, specificity: 62.1%) using a receiver operating characteristic (ROC) analysis and the calculation of the area under the curve (AUC) (0.721; 95% CI: 0.647–0.787; *p*  <  0.0001) (Figure 2). Using the above cut-off value, 79 out of 89 (89%) liver transplant recipients with TTV VL < 5.3 log copies/mL were responders after two vaccine doses, and 10 out of 89 (11%) were non-responders. Vice versa, 20 out of 37 (54.1%) patients with TTV VL ≥ 5.3 log copies/mL were responders and 17 out of 37 (45.9%) were non-responders after the third vaccine dose (Figure 3). Considering the whole population studied, only 16% (20/126) of cases are predicted incorrectly as non-responders and 8% (10/126) as responders. Thus, this predictive power was totally lost after the third vaccine dose.

Interestingly, when the covariates that were potentially able to influence vaccine responses in transplant patients (i.e., age, mycophenolate use, triple immunosuppression, time since transplantation, and TTV VL) were examined by regression analysis, TTV load was confirmed to be strongly associated with vaccine response as well as mycophenolate use (Table 3).

### 3.3. TTV VL and Anti-SARS-CoV-2 Neutralizing Antibodies Response

**Figure 4:**
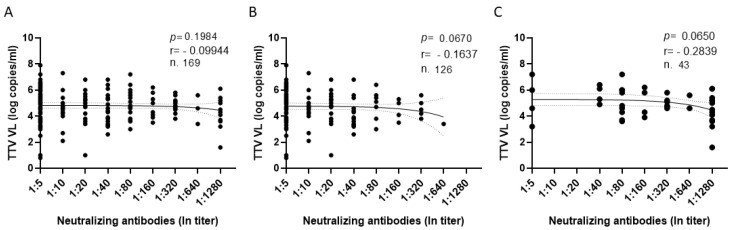
Correlation between TTV VL and neutralizing antibody titers measured totally (**A**), and after the second (**B**) and third (**C**) vaccine dose.

The correlation between TTV VL and titers of anti-SARS-CoV-2 neutralizing antibodies (nAbs) was also evaluated in all serum samples after the second and third vaccine doses. Overall, 105 out of 169 (62.1%) samples contained nAbs, including 66 of 126 (52.4%) and 39 of 43 (90.7%) samples after the second and third vaccine doses, respectively. Thirty-eight of 105 samples (36.2%) revealed nAbs titers ≥1:160 (12 and 26 samples after the second and third vaccine doses, respectively). Although liver transplant recipients with nAbs responses showed TTV VL slightly lower than LTRs with no nAb response (4.7 versus 5.1 log copies/mL, respectively), such a difference was not statistically significant. Additionally, TTV VL was not correlated with titers of nAbs produced after the second and third vaccine doses (Figure 4).

### 3.4. TTV VL and Cell-Mediated Immune Response

**Figure 5:**
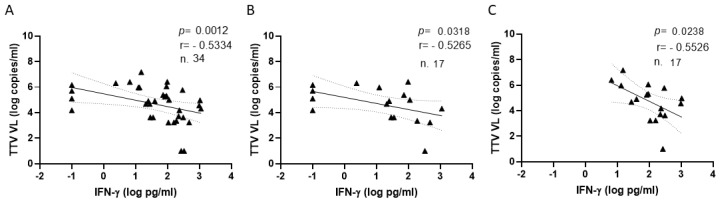
Correlations between TTV VL and levels of IFN-γ measured totally (**A**), and after the second (**B**) and third (**C**) vaccine dose.

Specific T-cell responses to S-peptides were evaluated in a subgroup of 17 liver transplant recipients after the second and third vaccine doses of the SARS-CoV-2 vaccine. After the second vaccination, 12 out of the 17 (70%) liver transplant recipients revealed a positive IFN-γ response with a median level of 84.3 pg/mL (IQR 26.2–298.5). A positive response was observed in 16 out of 17 patients (94%) at a median IFN-γ level of 133.5 pg/mL (IQR 50.9–290.2) after the third dose of SARS-CoV-2 vaccine. When pre-vaccine TTV VL were correlated with IFN-γ levels measured after the second and third doses of vaccine, statistically significant inverse correlations were found (Figure 5).

## 4. Discussion

Several studies have demonstrated higher loads of TTV in patients with immunosuppression compared to healthy control groups, and TTV VL kinetics in solid organ transplant are strongly associated with the strength and extent of immunosuppressive therapy [32,33]. Thus, the fact that levels of circulating TTV are a result of the immune function of the infected host is now consolidated. Previous studies showing that the measure of TTV DNA before the time of transplantation can be useful for predicting post-solid organ transplant complications further support the notion that the status of the immune system is strongly related to TTV VL [34]. Analogous to the studies noted above, we examined whether TTV load in the serum of liver transplant recipients could predict the response to the anti-SARS-CoV-2 vaccination and its breadth.

In similar studies carried out in solid organ transplant recipients, there was evidence that the response to immune stimulation with SARS-CoV-2 antigen may be related to TTV VL [35,36,37]. However, these studies examined TTV in kidney transplant recipients and lung transplant recipients, and no evidence has been reported so far investigating TTV loads and COVID-19 vaccine responses in liver transplant recipients. Again, until now, no relationship has been investigated between TTV VL and SARS-CoV-2 vaccination as evaluated in terms of nAbs production or cell-mediated response.

Some studies that have examined blood replication of TTV before immune stimulation with vaccine noted an association between VL and anti-Spike IgG response [28]. Among 197 kidney transplant recipients, Reindl-Schwaighofer et al. found that any doubling of TTV plasma levels at T0 predicted non-response (OR of 0.92) to homologous (ChAdOx1) or heterologous third dose in two-dose mRNA vaccine non-responders [38]. In a different study among 459 kidney transplant recipients, Solis et al. found that pre-vaccine TTV viral load > 6.2 log copies/mL was independently associated with non-response to two doses (OR = 6.17) as well as to three doses (OR = 3.62) [36]. In a third study from Vienna on 100 seronegative kidney transplant recipients, Graninger et al. found that plasma TTV loads at the time of the first vaccination were negatively associated with seroconversion after two-dose vaccination (OR 0.87) [39]. Similar results were achieved by Querido et al. in Portugal, who, in 114 adult kidney transplant recipients, found that TTV viremia at T0 ≥ 3.36 log copies/mL was associated with an unfavorable vaccine response (OR 5.40) after adjusting for age and estimated glomerular filtration rate at T0 [35]. Finally, Roberto et al. found that among 146 kidney transplant recipients, five logs of TTV viremia were the best threshold for predicting response to the second dose, and six logs were the best threshold for predicting response to the third dose of the COVID-19 vaccine [40].

In lung transplant recipients, Hoek et al. showed that lower TTV viremia pre-vaccination was significantly associated with a better response to the first mRNA-1273 vaccine dose (odds ratio (OR): 0.54; *p* < 0.0001) [37]; Gallais et al. reported an adjusted OR of 17.8 (*p* = 0.001) and response to the third BNT162b2 vaccine dose for pre-vaccine TTV viral load ≥6.2 log copies/mL [41]. Roberto et al. found, in 26 lung transplant recipients, that five logs of TTV viremia were the best threshold for predicting response to the second dose, and six logs were the best threshold for predicting response to the third COVID-19 vaccine dose [40]. Compared to other solid organ transplant recipients, lung transplant patients receive higher levels of immunosuppression and, accordingly, have reduced COVID-19 vaccine responses [12].

In our previous studies on healthy individuals, we demonstrated that an efficient immune response to the influenza vaccine (30 days after vaccination) exists in subjects with a median baseline TTV VL of 3.8 log copies/mL. In addition, when measured at day 90 after vaccination, more than 80% of individuals with a median baseline TTV VL of 4.1 log copies/mL have a response to the hepatitis B vaccine [42]. TTV load may therefore be used to determine the optimal vaccination schedule in patients under maintenance of immunosuppression as suggested by Rezahosseini et al. [43], and the evidence published so far is strengthening this hypothesis. These observations suggest the existence of a continuum: the higher the immunosuppression, the better the correlation between basal TTV loads and antibody responses induced by vaccination.

The present study offers further information on this matter. First, it profiles the TTV VL in liver transplant recipients before SARS-CoV-2 vaccination and demonstrates that load size highly reproduces the extent of post-vaccine antibody response in this group of immunocompromised patients. Second, the study attempts to correlate TTV viremia with additional indicators of effective vaccine response, particularly nAb titers and cell-mediated response. Interestingly, in our study we also found a correlation between TTV VL and cell-mediated immune response as measured by IFN-γ release assays, thus suggesting the potential role of the virus as a biomarker of the global immunological response to vaccination. However, additional studies using different methodologies are needed for a more focused evaluation of this correlation. The lack of TTV correlation with nAb titers induced by SARS-CoV-2 vaccination may depend on several factors, some of which are due to the relatively poor extent of the measured antibody levels that could impact ascertaining possible correlations. Other studies are warranted to shed light on this aspect. Third, we corroborated the usefulness of TTV VL monitoring to predict the response to anti-SARS-CoV-2 vaccination, suggesting a threshold level of 5.3 log copies of TTV.

Certainly, at this stage, to consolidate the potential predictive role of TTV, further and larger observational studies are required. However, TTV viremia-driven vaccine boosts represent an interesting opportunity to improve the rate of response in immunocompromised individuals. For this purpose, to assess utility in association with further correlates of vaccine response, the integration of a pilot trial within the Horizon2020-funded “TTV Guide Tx” project (https://www.ttv-guide.eu/, accessed on 26 October 2023) [22] should be guaranteed.

To date, in solid organ transplant recipients, advanced age, mycophenolate use, triple immunosuppression, and more advanced stages of organ failure are the only reliable predictors of response to COVID-19 vaccines [37,38,41,44,45,46]. While there are discordant results for time since transplantation [37,38].

In conclusion, in the setting of solid organ transplant, TTV VL is quickly becoming a marker of immune function with the ability to not only predict opportunistic infections and graft rejections, but also the response to vaccination. If TTV has the potential to be used as a vaccine response marker in other larger population settings, that must be furtherly investigated.

## Figures and Tables

**Table 1:** Basal characteristics of the study patients, grouped by vaccine dose.

Parameter	Liver Transplant Recipients 1st + 2nd Dose (n. 126)	Liver Transplant Recipients 3rd Dose (n. 43)
Age in years—mean ± SD	63.2 ± 9.3	63.6 ± 7.7
Male—n. (%)	103 (81.8)	38 (88.4)
HIV positive—n. (%)	0 (0)	3 (7.0)
BMI > 30—n. (%)	24 (19.0)	9 (20.9)
Diabetes mellitus—n. (%)	34 (27.0)	12 (27.9)
Cancer—n. (%)	7 (5.5)	4 (9.3)
Years since transplantation—median (IQR)	7.0 (4.0–12.0)	7.0 (3.0–14.0)
Patients with ≤6 years since transplantation—n. (%) ^a^	56 (44.4)	20 (46.5)
Patients with >6 years since transplantation—n. (%)	70 (55.6)	23 (53.5)
Therapy—n. (%)		
Tacrolimus	117 (92.8)	39 (90.7)
Everolimus	16 (12.7)	5 (11.6)
Mycophenolate mofetil	70 (55.5)	21(48.8)
Steroids	4 (3.2)	3 (7.0)
Pre-dose TTV DNA positive—n. (%)	124 (98.4)	43 (100)
Pre-dose Log copies/mL TTV VL—median (IQR)	4.7 (3.9–5.5)	4.9 (4.3–5.8)

^a^ Six years was chosen as the cut-off time for classifying patients as long-term survivors with a higher immunological response according to [4]. Abbreviations: BMI, body mass index; IQR, interquartile range; SD, standard deviation; TTV VL, TTV viral load.

**Table 2:** Characteristics of the study patients, grouped by anti-SARS-CoV-2 dose response.

Parameter	2nd Vaccine Dose			3rd Vaccine Dose	
	Responders ^a^ (n. 99, 78.6%)	Non-Responders (n. 27)	*p* Value	Responders (n. 41, 95.3%)	Non-Responders (n. 2)
Age in years—median (IQR)	63 (59–70)	64 (58–69)	NS	65 (58–70)	64 (61–67)
Male—n. (%)	84 (84.8)	19 (70.4)	NS	36 (87.8)	2 (100)
HIV positive—n. (%)	0 (0)	0 (0)	NS	2 (4.9)	1 (50)
BMI > 30—n. (%)	22 (22.2)	2 (7.4)	NS	9 (21.9)	0 (0)
Diabetes mellitus—n. (%)	27 (27.3)	7 (25.9)	NS	12 (29.3)	0 (0)
Cancer—n. (%)	7 (7.1)	0 (0)	NS	4 (9.8)	0 (0)
Years since transplantation—median (IQR)	8.0 (5.0–12.0)	3.0 (1.0–9.0)	0.0028	7.0 (4.0–14.5)	2.0 (1.0–3.0)
Patients with ≤6 years—median (IQR; n.)	4.0 (3.0–5.0; 39)	2.0 (0.5–3.0; 17)	<0.0001	3.5 (2.0–5.0, 18)	2.0 (1.0–3.0; 2)
Therapy—n. (%)					
Tacrolimus	92 (92.9)	25; 92.6	NS	37; 90.2	2; 100.0
Everolimus	12 (12.1)	4 (14.8)	NS	4 (9.8)	1 (50)
Mycophenolate mofetil	45 (45.5)	25 (92.6)	<0.0001	20 (48.8)	1 (50)
Steroids	0 (0)	4 (14.8)	<0.0001	2 (4.9)	1 (50)
Pre-dose TTV DNA positive—n. (%)	97 (98.0)	27 (100)	NS	41 (100)	2 (100)
Pre-dose Log copies/mL TTV VL—median (IQR)	4.6 (3.8–5.2)	5.6 (4.7–6.3)	<0.0001	4.9 (4.2–5.8)	5.3 (4.6–6.0)

^a^ Responders and Non-responders were patients with anti-SARS-CoV-2 RBD titers ≥ 7.1 BAU/mL [median 2.1 (IQR: 1.0–2.8) after second dose, and 3.4 (IQR: 2.7–3.8) after third dose] and <7.1 BAU/mL, respectively. Abbreviations: BMI, body mass index; IQR, interquartile range; NS, not significant; TTV VL, TTV viral load.

**Table 3:** Odds ratios (ORs) for having vaccine response for 5 independent variables.

Variable	OR	OR (95% CI)	*p* Value
Age	1.0	0.9–1.0	NS
Mycophenolate use	9.9	2.7–36.6	0.001
Triple immunosuppression	6.0	0.6–64.5	NS
TTV VL pre-vaccination ≥ 5.3 log copies/mL	4.8	1.8–12.9	0.002
Years since transplantation ≤ 6 years	1.4	0.5–3.9	NS

Abbreviations: CI, Confidence Interval; NS, not significant; TTV VL, TTV viral load.

## Data Availability

The data that support the findings of this study are available from the corresponding author upon reasonable request.
